# New Approach for Untangling the Role of Uncommon Calcium-Binding Proteins in the Central Nervous System

**DOI:** 10.3390/brainsci11050634

**Published:** 2021-05-14

**Authors:** Krisztina Kelemen, Tibor Szilágyi

**Affiliations:** Department of Physiology, Doctoral School, Faculty of Medicine, George Emil Palade University of Medicine, Pharmacy, Science, and Technology of Targu Mures, 540142 Târgu Mureș, Romania; tibor.szilagyi@umfst.ro

**Keywords:** calcium-binding proteins, central nervous system, human protein atlas, in situ hybridisation database, transcriptome database

## Abstract

Although Ca^2+^ ion plays an essential role in cellular physiology, calcium-binding proteins (CaBPs) were long used for mainly as immunohistochemical markers of specific cell types in different regions of the central nervous system. They are a heterogeneous and wide-ranging group of proteins. Their function was studied intensively in the last two decades and a tremendous amount of information was gathered about them. Girard et al. compiled a comprehensive list of the gene-expression profiles of the entire EF-hand gene superfamily in the murine brain. We selected from this database those CaBPs which are related to information processing and/or neuronal signalling, have a Ca^2+^-buffer activity, Ca^2+^-sensor activity, modulator of Ca^2+^-channel activity, or a yet unknown function. In this way we created a gene function-based selection of the CaBPs. We cross-referenced these findings with publicly available, high-quality RNA-sequencing and in situ hybridization databases (Human Protein Atlas (HPA), Brain RNA-seq database and Allen Brain Atlas integrated into the HPA) and created gene expression heat maps of the regional and cell type-specific expression levels of the selected CaBPs. This represents a useful tool to predict and investigate different expression patterns and functions of the less-known CaBPs of the central nervous system.

## 1. About Ca^2+^ Briefly

The Ca^2+^ ion plays an essential role in cellular physiology. It has two major functions: as an ion it affects the membrane potential, and as a second messenger it activates several intracellular mechanisms, e.g., the contraction of the myocardium, hormone secretion by endocrine cells, enzyme activation, degranulation of various white blood cells, as well as neurotransmitter release from the synaptic terminals. Besides these rapid effects, calcium has longer-lasting effects too (like long-term potentiation), and influences even gene transcription, cell proliferation and differentiation.

In the resting state of a cell the concentration of free calcium in the cytoplasm varies between 10–100 nmol/L, more than a 1000-fold lower than that in the blood. A functionally optimal concentration is achieved by the fine-tuned balance of input-output mechanisms. In general, the intracellular increase in calcium concentration can be attributed to two main mechanisms. On one hand, the Ca^2+^ ions can enter the cell from the extracellular space through the ion channels of the cell membrane that can be gated by the membrane potential, ligand binding or other factors. On the other hand, Ca^2+^ is released from internal stores, such as the endoplasmic reticulum (named sarcoplasmic reticulum in the muscle cells), mitochondria or calcium buffers. The release of Ca^2+^ from the internal stores is led by Ca^2+^ itself or by a group of distinct messengers. Voltage-gated calcium entry takes place in electrically excitable cells whereas in non-excitable cells the intracellular calcium level increase is mainly the result of release from the internal stores. The main mechanisms for decreasing the intracellular Ca^2+^ concentration are the different exchangers and pumps, which remove Ca^2+^ from the cytosol. Their importance varies depending on the cell type. These processes transport calcium against its electro-chemical gradient and, therefore, are energy dependent. Calcium can be pumped into the organelles (intracellular Ca^2+^ stores), or it can be pumped to the extracellular space via plasma membrane calcium pumps or by indirect active transport achieved by exchanger proteins. The intracellular Ca^2+^ concentration is also dependent on the dynamic binding to different calcium-binding proteins.

An excessive amount of intracellular calcium can lead to cell damage or even cell death. By this mechanism, over-excitation of neural circuits can cause excitotoxicity. Therefore, the fine-tuning of calcium levels by several receptors, calcium-channels, calcium pumps and exchangers, calcium-buffers and numerous calcium-binding proteins (CaBPs) is of utmost importance.

## 2. Calcium in Neurons

Neurotransmitter release is closely correlated to calcium levels. Calcium ion channel density is high at active zones in the presynaptic terminals, opposite the post-synaptic receptors. Calcium ions do not diffuse far from their site of entry because they are immediately buffered by calcium-binding proteins [1]. Therefore, calcium influx generates a local rise in calcium concentration at the site of the active zones. In all synapses neurotransmitter release has a non-linear correlation with Ca^2+^ influx, meaning that a twofold increase in Ca^2+^ levels can produce a 16-fold increase of the amount of neurotransmitter released [2]. By fine-tuning the calcium levels in the presynaptic terminal, throughout the duration of the action potential, the neurotransmitter release is also regulated, therefore the synaptic transmission is also affected [3].

Calcium, as a second messenger, carries signals throughout the nerve cell as a reaction to membrane depolarization. Therefore it is transferring information on neuronal activity status both locally, for example in a dendritic spine or a small dendritic segment, and all over within the neuron, for instance to increase energy metabolism [4]. Changes in Ca^2+^ levels are often restricted to specific regions of the cytosol, allowing different processes (like exocytosis of a secretory vesicle) to happen locally, without affecting other processes elsewhere in the cell [5]. A crucial function of calcium is to regulate activity-dependent signalling. By feed-back and feed-forward mechanisms, calcium signalling reinforces relevant synaptic connections, eliminates unnecessary connections and evades overexcitation, therefore it controls neuronal excitability [6] and it is involved in the mechanisms of learning and memory.

## 3. The Role of Ca^2+^ Binding

The cytosolic Ca^2+^ concentration can transiently increase by 10–100 fold, due to the signal-induced release from the endoplasmic reticulum or influx from the extracellular medium by calcium channels. The sudden cytosolic change in calcium level is detected by certain Ca^2+^-binding proteins [5]. These calcium binding proteins regulate Ca^2+^ concentration but they are also regulated by the intracellular amount of calcium. The concentration of free cytosolic calcium in a resting cell is low due to the uptake into the endoplasmic reticulum and/or the continuous transport out of the cell, orchestrated by ATP-fuelled pumps. Complementing these main input–output mechanisms, when calcium buffers become loaded with calcium, they serve as fine-tuning agents of the spatial and temporal properties of calcium signals. These proteins influence both the amplitude and the recovery time of calcium transients [7]. Furthermore, CaBPs possibly directly or indirectly facilitate sensitization or desensitization of calcium channels and may cut off additional calcium entry into the cell [8]. 

**Figure 1:**
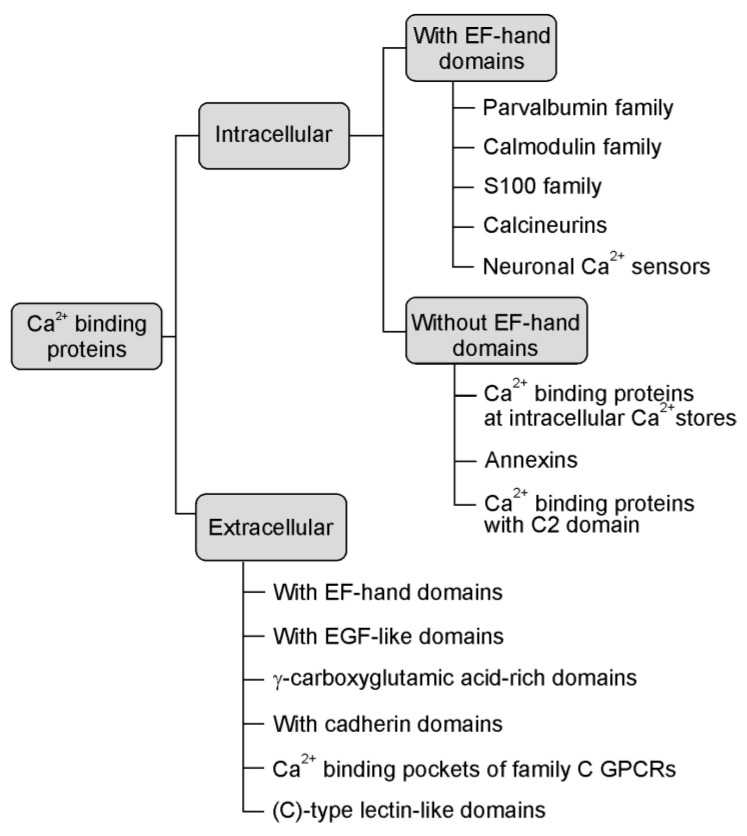
The main types of calcium-binding proteins (CaBPs) [9].

Ca^2+^ binding proteins are a heterogeneous and extensive group of proteins (see Figure 1, based on Elies et al. [9], that are engaged in many cellular and extracellular functions from calcium homeostasis to calcium signalling pathways [10]. The calcium signal is decoded by various calcium-binding motifs. These are present in the specialised calcium-buffer and calcium-sensor proteins that couple changes in calcium concentration to a wide variety of functions depending on their disposition and calcium source [11]. Despite the fact that they have various structures and properties, they selectively and reversibly bind calcium in specific domains, the kinetics of this interaction frequently being very fast [12]. 

The rise in cytosolic calcium is sensed especially by EF-hand family CaBPs. The large family of EF-hand domain-containing proteins comprises of various important and ubiquitously expressed CaBPs, like calmodulin [12,13], but also several proteins with functions that we are just beginning to understand. 

## 4. The EF-Hand Calcium-Binding Proteins

**Figure 2:**
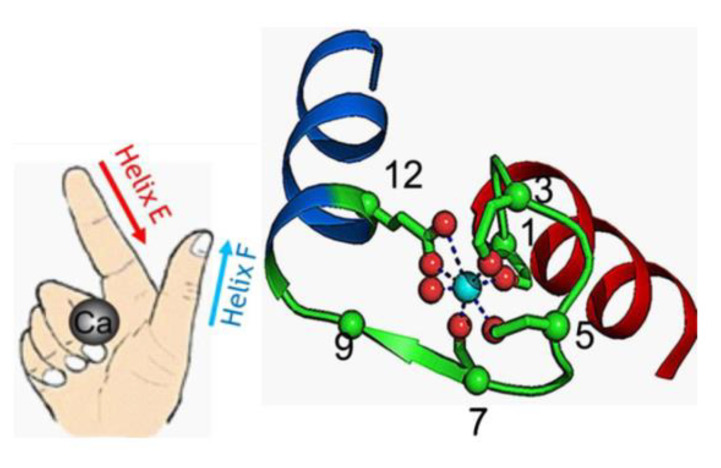
The helix-loop-helix EF-hand Ca^2+^-binding motif based on Zhou et al., 2009 (Wikimedia commons licence). The conformation of the helixes resembles a spread thumb and forefinger of a human hand. In the representative 3D model of a typical canonical EF-hand motif from calmodulin, the Ca^2+^ ion is integrated within the loop, which includes seven oxygen atoms from the sidechain carboxyl or hydroxyl groups (loop sequence positions 1, 3, 5, 12), a main chain carbonyl group (position 7), and a bridged water (via position 9) [16]. Figure copyright permission obtained.

Among the different types of calcium-binding proteins, the EF-hand CaBPs represent the largest family, for example, humans express approximately 250 EF-hand containing proteins [14]. This family includes proteins consisting of one or more EF-hand domains and possessing various functions like calcium buffering in the cytosol or signal transduction between cellular compartments. The term EF-hand is a descriptive one. It not only refers to the molecular structure of this calcium-binding domain but also to its motion that the binding of calcium can generate [15]. The EF-hand motif itself is composed of a highly conserved sequence of 12 amino acids which can chelate a single Ca^2+^ ion, surrounded by two α-helices. These two helices are bound by the short loop in such a distinct way, that the helix-loop-helix motif can be seen as the spread thumb and forefinger of a human hand [5] (see Figure 2). The calcium ions are coordinated within the loop and the affinity for Ca^2+^ is a determining factor for the function of the protein. In some cases the loop can accommodate Mg^2+^ as well [15].

Frequently, EF-hand motifs occur in adjoining pairs, for instance, calmodulin and troponin C contain 4 EF-hand motifs, whereas parvalbumin contains only 2 EF-hand motifs [12]. Calcium ions bind to EF-hand domains with different affinities in a range of 10^−6^ M to 10^−3^ M, which allows them to fulfil their diverse biological functions [11,17].

From a functional point of view calcium-binding proteins can be divided into two major groups. First, there are the Ca^2+^ sensor proteins/signal modulators, which have a dissociation constant (K_d_) K_d_ > 0.1 μM (e.g., calmodulin family, S100 families, synaptotagmin). These proteins have relatively low affinity to calcium and undergo significant conformational changes upon binding calcium in order to further interact with specific downstream targets, thereby switching their activities on or off [18]. A great deal of what we know about the mechanism of action of Ca^2+^ sensors is based on the structural analysis of calmodulin. If calcium sensors are present in high concentration, they can act as calcium buffers as well [19].

The second main group comprises of the Ca^2+^ buffers which have high affinity, K_d_ < 0.1 μM, that are thought to mainly chelate calcium (e.g., parvalbumin) [18,20]. In this case the binding of calcium and, therefore, the dispersion of local calcium does not cause major modification of the protein structure. They do not directly influence the activity of other macromolecules [21], instead, they moderate calcium transients. They modulate the shape and/or duration of Ca^2+^ signals and help maintain Ca^2+^ homeostasis. Their main function is to control free calcium in the cell. During resting-state calcium buffers are mainly free of calcium, but if calcium levels rise, these proteins will modulate the spatiotemporal aspects of calcium signals. The way in which calcium transients are affected is also influenced by the intracellular distribution and mobility of these calcium-buffers [22]. By modifying the calcium concentration, calcium buffers can indirectly affect neuronal excitability and synaptic plasticity [21]. 

There is no distinct boundary between the function of calcium sensors and buffers, seeing that the activity of these proteins is conditioned by their local concentration and the availability of interacting partners in signalling networks [23].

## 5. The Regional Arrangement of the EF-Hand CaBPs Throughout the Central Nervous System (CNS)

The distribution of EF-hand CaBPs throughout the central nervous system (CNS) is not homogeneous. Some of them are expressed ubiquitously, whereas others have distinct expression patterns, they can be limited to specific brain regions, subtypes of neurons or glial cells or they can appear in limited time intervals during the development of the CNS. Members of the calmodulin calcium-binding family are frequently mentioned as ubiquitously expressed proteins in all eukaryote cells. As an opposite example, we can mention the specific expression pattern of CaBPs like secretagogin, which is expressed by amacrine cells and rod photoreceptors of the retina [24], and neurons of the olfactory bulb [25,26]. Recently other CaBPs, the N-terminal EF-hand calcium-binding proteins (NECAB) 1 and 2 were shown to be expressed predominantly by CB_1_/CCK-positive interneurons throughout the isocortex, hippocampal formation and basolateral amygdala complex. Furthermore, they exhibit different subcellular distribution in the CB_1_/CCK-positive GABAergic interneurons [27].

## 6. The “Classical” CaBPs of the CNS 

CaBPs were regarded initially as immunohistochemical markers of specific cell types in different regions of the CNS. Parvalbumin, calbindin and calretinin are the most often used CaBPs for this purpose, especially for the identification of inhibitory local circuit neurons [28,29]. Sometimes their presence can predict cellular excitability and the fashion of neurotransmitter release following electrical stimuli. As these proteins have an essential role in cellular physiology, they deserve our attention, not just as cellular markers. Their complex and often interlaced roles in calcium homeostasis, in intracellular signalling, and also their alterations in brain diseases should be elucidated.

In the last two decades, a tremendous amount of information has been published about CaBPs. It is beyond the scope of our review to give a comprehensive overview of all CaBPs and Ca^2+^-dependent signalling cascades. We will focus on the neuronal CaBPs, their function and distribution in the central nervous system by systematically analysing publicly available, high-quality RNA-sequencing and in situ hybridization (ISH) databases. This allows us to better understand the specific spatiotemporal distribution and function of genes expressed in the brain. By summarising quantitative regional and cell-specific data in a visually clear way, our review aims to find relevant information regarding the functionally important CaBPs in the brain.

## 7. Search for New CaBPs. Detailed Analysis of RNA-Seq and ISH Databases

Through extensive examination of the Uniprot (www.uniprot.org accessed on 12 May 2021) and National Center for Biotechnology Information (NCBI Gene, www.ncbi.nlm.nih.gov/gene accessed on 12 May 2021) databases, Girard et al. found that the EF-hand family comprises in mouse at least 249 putative members [30]. With the help of Uniprot database and the Allen Brain Atlas (ABA, www.brain-map.org accessed on 12 May 2021) they created a meticulous list of the EF-hand super-family in the mouse genome. The functions of these genes were given using Gene Ontology, including roles like participation in muscle homeostasis, leucocyte adhesion, regulation of pH, apoptosis, etc. For the complete list of their integrated functions according to Gene Ontology terms see Table 1, adapted from Girard et al.

Out of the pool of various functions we carefully selected those CaBPs that are connected to information processing and/or neuronal signalling, have Ca^2+^-buffer activity, Ca^2+^- sensor activity, modulator of Ca^2+^ channel activity or yet unknown function (Table 1), furthermore they show detectable gene expression in the brain. In this way we created a gene function-based selection of the calcium-binding proteins.

**Figure 3:**
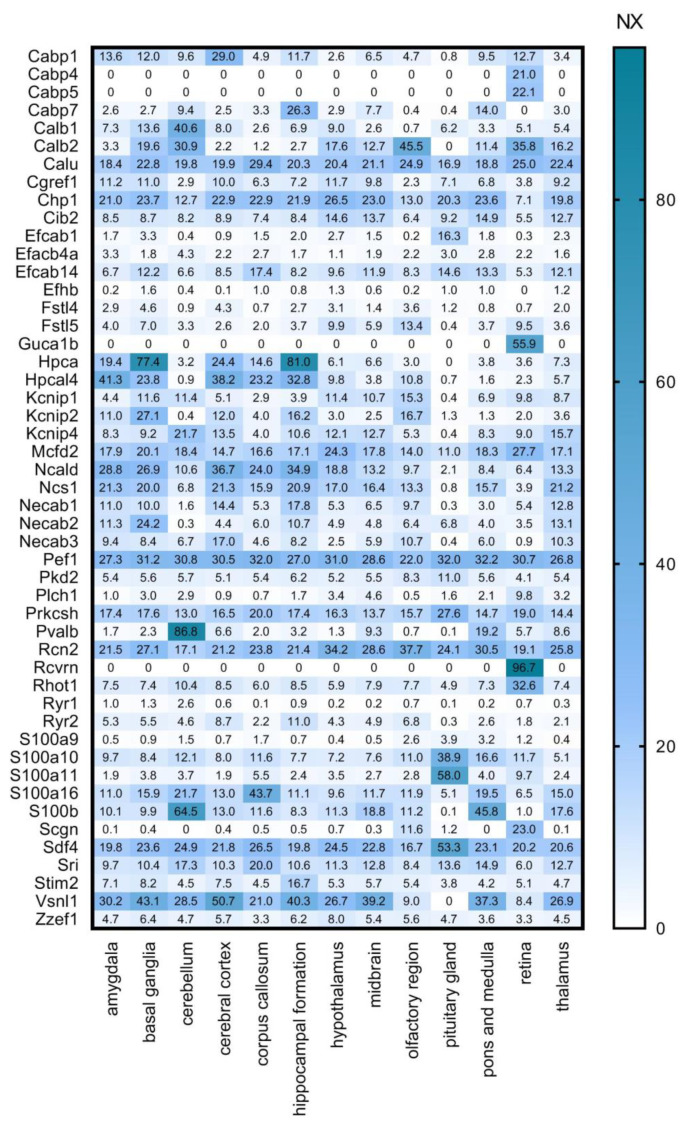
Regional expression levels of the selected CaBP genes. Mouse gene data from Human Protein Atlas (HPA) transcriptomics analysis is shown as NX values in different brain regions. Numerical data is presented in Supplementary Materials Table S1.

We cross-referenced our calcium-binding protein list with several other databases. First, we used the Human Protein Atlas (HPA, www.proteinatlas.org, accessed on 29 April 2020, [31,32]) Brain Atlas transcriptomics analysis performed on multiple brain regions (Figure 3). In this dataset, transcript expression levels are summarized per gene in 13 brain regions based on RNA-seq transcripts per million (TPM) and protein-coding transcripts per million (pTPM). The TPM values were TMM (trimmed means of M values) normalized between all samples respectively, and then each gene was Pareto scaled. The normalized expression (NX) calculation is based on pTPM of the individual samples. The Human Protein Atlas version 19.3 and Ensembl version 92.38 were used for analysis. To shine a light on the regional expression levels of the selected CaBPs we created the following gene expression heat maps.

**Figure 4:**
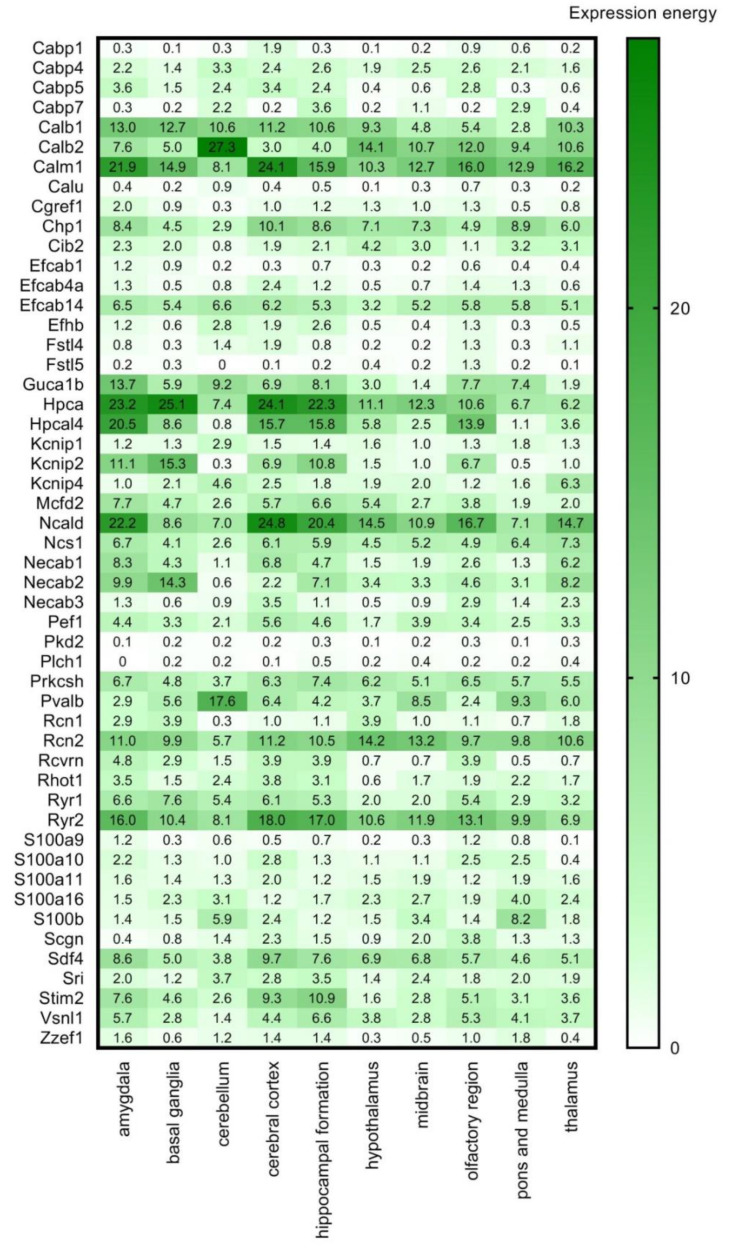
Transcript expression levels in 10 brain regions based on in situ hybridisation. RNA Allen mouse brain region gene data of the selected CaBPs from in situ hybridization (ISH) analysis is represented by the expression energy value. Numerical data are presented in Supplementary Materials Table S2.

As a next step we used the RNA Allen mouse brain region gene data (obtained from the Allen Brain Atlas, ABA) integrated into the HPA, which contains transcript expression levels summarized per gene in 10 brain regions based on in situ hybridisation (ISH) in the adult mouse brain. This ISH data provides spatial expression data on a single-cell level (Figure 4). The expression energy shown in Figure 4, based on ISH, is defined as the sum of expression pixel intensity divided by the sum of all pixels in division [33].

High overlap and complementarity can be observed between the HPA transcriptomics and the ISH databases (Figure 3 and Figure 4). 

In order to obtain more specific information about the distinct expression patterns of the aforementioned proteins, we used a cell-type-specific analysis, RNA-seq results of selected cells using immunopanning from the Brain RNA-seq database (www.brainrnaseq.org, accessed on 12 May 2020). Whilst creating this database, Ye Zhang et al. (2014) [34] purified the major cell types in the adult mouse brain, namely neurons, astrocytes, various maturation states of oligodendrocytes, microglia and endothelial cells. For all cell types, one biological replicate consisted of pooled cells from a litter of 3–12 mice. To create a transcriptome database by RNA sequencing, two replicates of pooled animals for each cell type were sequenced. Expression level estimation is reported as fragments per kilobase of transcript sequence per million mapped fragments (FPKM) value together with confidence intervals for each sample.

**Figure 5:**
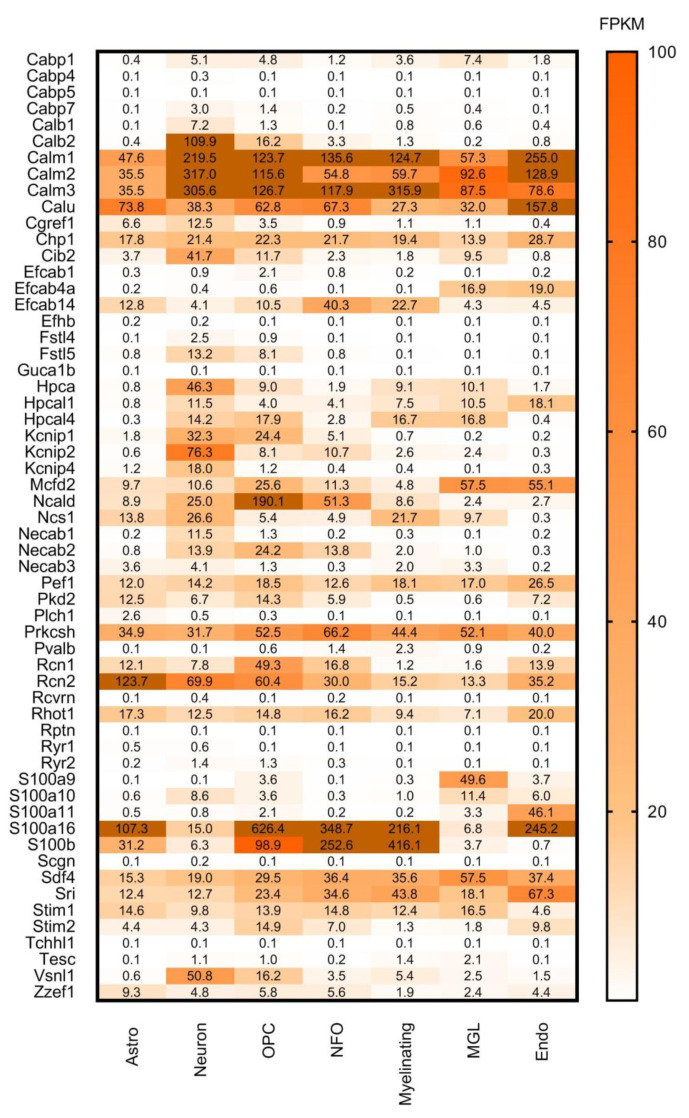
Cell-type specific gene expression levels of selected CaBPs from the Brain RNA-seq database. Expression level estimation is reported as fragments per kilobase of transcript sequence per million mapped fragments (FPKM). Astro: astrocytes, OPC: oligodendrocyte progenitor cells, NFO: newly formed oligodendrocytes, Myelinating: myelinating oligodendrocyte, MGL: microglia/macrophage, Endo: endothelial. Numerical data are presented in Supplementary Materials Table S3.

This dataset allows us to visualize the expression levels of the selected CaBPs in the major cell types in the adult mouse brain (Figure 5).

**Figure 6:**
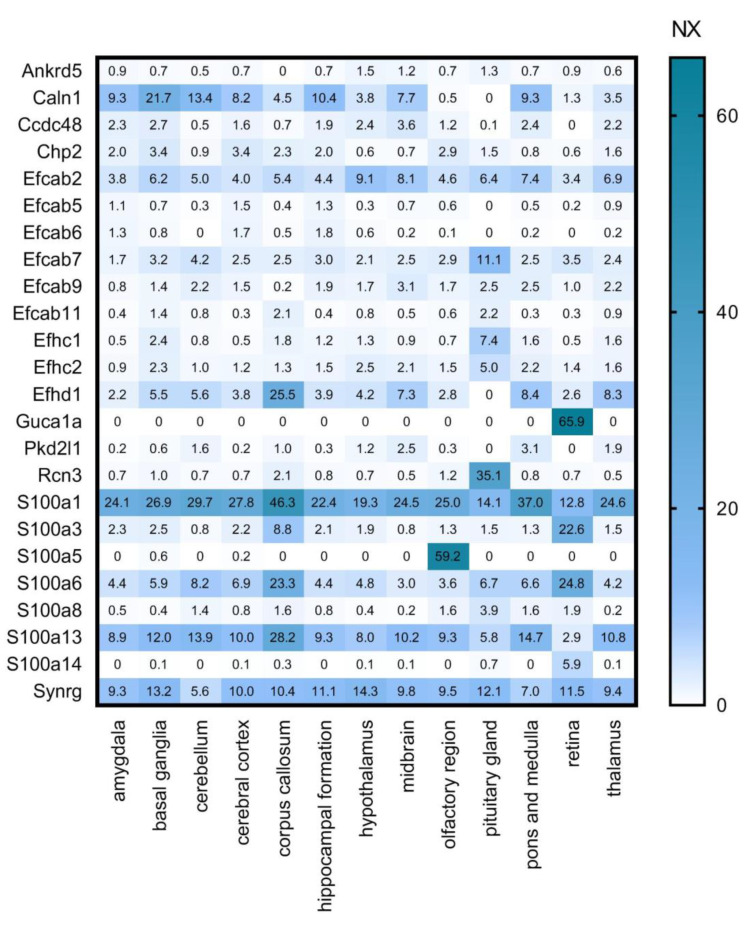
Mouse regional gene data of CaBPs with lower expression in the CNS by Girard database. Transcriptomics analysis from HPA showing normalized expression (NX) values of genes in different brain regions. Numerical data are presented in Supplementary Materials Table S4.

**Figure 7:**
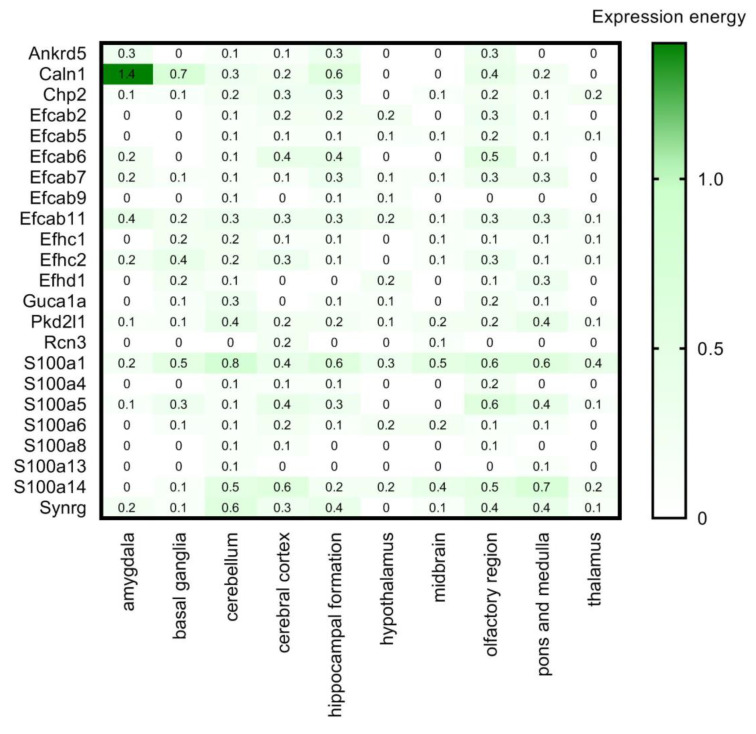
Transcript expression levels of CaBPs with lower expression in the CNS by Girard database in 10 brain regions, based on in situ hybridisation. RNA Allen mouse brain region gene data is represented by the expression energy value. Note the different color scale from Figure 4. Numerical data are presented in Supplementary Materials Table S5.

**Figure 8:**
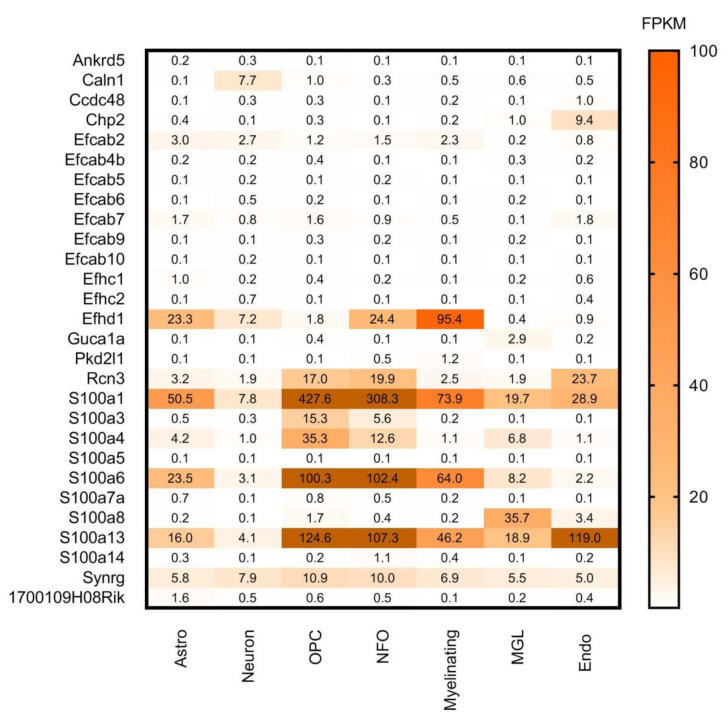
Cell-type specific gene expression levels of CaBPs with lower expression in the CNS by Girard database. Expression level estimation from Brain RNA-seq database is reported as fragments per kilobase of transcript sequence per million mapped fragments (FPKM). Astro: astrocytes, OPC: oligodendrocyte progenitor cells, NFO: newly formed oligodendrocytes, Myelinating: myelinating oligodendrocyte, MGL: microglia/macrophage, Endo: endothelial. Numerical data are presented in Supplementary Materials Table S6.

In the analysis of Girard et al. some genes are mentioned as ‘no expression detected in the brain (n.e.d.)’ or ’no data available in the ABA database (n.d.)’, but when we cross referenced these genes with the HPA database, several of them were found. These genes are presented in Table 2 and Figure 6, Figure 7 and Figure 8.

The aim of our work was to provide a condensed database and visual presentation of data scattered over several primary sources. Using the color-coded figures, it is easier to spot relevant expression patterns of different CaBP genes. These figures can help to find which genes have the highest expression level in a given region, and which proteins occur in the same place. It is also possible to combine information on their distribution patterns in different brain regions and cell types. Next we present a few examples regarding the application of our databases and data visualisation.

In Figure 3 it is striking that **Cabp4, Cabp5, Guca1b, Rcvrn** are expressed exclusively in the retina, and also **Plch1, Rhot1** and **Scgn** have a much stronger expression here than in other parts of the CNS. This group can, therefore, be considered as important retinal calcium-binding proteins. This observation is consistent with the literature: CaBP4 is localised in photoreceptor synaptic terminals and is essential for neurotransmission between photoreceptors and bipolar cells as part of the Cav1.4 channel complex in the retina [35]; CaBP5 in mice is expressed in type 5 ON-cone bipolar cells, and in type 3 OFF-cone bipolar cells as well as in rod bipolar cells [36,37,38]; Guca1b is important for rod cell recovery after light exposure, by stimulation of guanylate cyclases in these photoreceptors [39]; Secretagogin plays an essential role in synapse maturation and in mouse, rat, and rabbit retina it is expressed in subtypes of cone bipolar cells, but cannot be detected in the rod bipolar cells [40]; Recoverin is thought to be a calcium sensor in retinal rod cells that can control the lifetime of photoexcited rhodopsin by inhibiting rhodopsin kinase [41,42]; and last but not least Plch1 has been shown to be expressed in mouse and human retina as well [43].

Proteins like **Calm1, Calm2, Calm3, Calu, Chp1, Pef1, Prkcsh, Rcn2, Sdf4, S100a16,** and **S100b** are highly and ubiquitously expressed by all the examined cell types: astrocytes, neurons, oligodendrocyte progenitor cells, newly formed oligodendrocytes, myelinating oligodendrocyte, microglia/macrophage, endothelial cells (Figure 5). If these proteins are expressed by all cell types, we expect that they should be present in all regions of the brain. Cross-referencing these findings with Figure 3 and Figure 4, it can be observed that these proteins are present indeed in all the representative brain regions and almost at the same expression level. Some of these genes (Calm1, Calm2, Calm3, Prkcsh, S100a16 and S100b) code proteins that have Ca^2+^ sensor/buffer activity. The function of the others (Calu, Chp1, Pef1, Rcn2, Sdf4) is yet unknown, but since they are similarly scattered through the brain, we may conclude that they might have fundamental functions in the cell’s calcium homeostasis as well.

In Figure 5 it is conspicuous that hippocalcin **(Hpca),** voltage-gated potassium channel interacting protein **(Kcnip2)** and visinin-like protein 1 **(Vsnl1)** are mainly expressed by neurons. **Kcnip2** shows high expression levels in the basal ganglia, hippocampal formation, amygdala and cerebral cortex but it is less expressed in the cerebellum. This Ca^2+^ sensor protein is one of the four K^+^ channel interacting proteins (Kcnips1–4), which interacts with and modulates the activity and trafficking of Kv4 potassium channels [44]. It is a fundamental component of the endogenous A-type Kv channel complex and being an auxiliary subunit of this channel it is confined to intracellular membranes and the cell membrane [45,46,47]. In the hippocampus and neocortex Kcnip2 was found in association with the Kv4.2 subunit of the Kv4 potassium channel, mainly expressed in the apical and basal dendrites of glutamatergic pyramidal neurons [47,48,49]. The co-presence of Kcnip2 and Kv4 subunits indicates that this CaBP is likely to play a major role as modulator of somatodendritic excitability [47]. Furthermore, Kcnip2 expression is linked to the overall survival of glioblastoma patients, thereby gaining clinical significance [50].

**Hippocalcin** is a member of the neuronal calcium sensor protein family, dominantly expressed in the pyramidal cell layer of the hippocampus [51], moderately in the dentate granule cells and the pyramidal cells of cerebral cortex layers II–VI and weakly in the large neuronal cells of the caudate-putamen. It is mainly localized in the cytoplasm and plasma membrane of dendrites and soma [52]. Palmer et al. described its important calcium-sensing role in NMDAR (N-methyl-D-aspartate receptor)-mediated hippocampal LTD (long-term depression), where it couples NMDAR activation to the endocytosis of AMPARs (α-amino-3-hydroxy-5-methyl-4-isoxazolepropionic acid receptor) [53]. It was shown that hippocalcin as a diffusible calcium sensor is the key intermediate between calcium and potassium channels that mediate a slow afterhyperpolarization current [54]. This suggests that Hpca and Kcnip2 might have complementary roles in pyramidal cell excitability.

**Visinin like protein 1 (Vsnl1)** is also mainly expressed by neurons and mostly in the cerebral cortex, hippocampal formation, amygdala, hypothalamus, thalamus, midbrain and cerebellum, which is consistent with the observation that VSNLs show a widespread but distinct expression pattern primarily in nerve cells [55]. More detailed studies show strong expression levels in subpopulations of calbindin-D28K and calretinin- positive GABAergic interneurons in all hippocampal regions of the rat brain, but Vsnl1 was detected in principal cells as well [56]. Vsnl1 is a Ca^2+^ sensing protein and it influences dendritic growth, cyclic nucleotide signalling, and nicotinic modulation of neuronal network activity, and therefore it regulates synaptic plasticity [57]. It also plays a functional role in integrating the cytosolic calcium concentration and the oxidative status of the cell [58]. Visinin-like protein 1 has been identified as a biomarker for Alzheimer’s disease [59], and it has been shown to play an important part in the development of amyotrophic lateral sclerosis [58,60].

These few examples show that the databases we created can be used to further predict and investigate different expression patterns of the little known CaBPs. The various kinetic and buffering properties of CaBPs help modulate the synaptic responses and the excitability of neurons, and therefore they are adapted to the specific needs of the cell populations that express them. The kinetics of calcium binding and the dissociation constant (Kd) varies between different CaBPs. For instance, the calcium buffering capacity of motoneurons and adrenal chromaffin cells is many folds lower than that of the Purkinje neurons. The low buffering capacity of motoneurons allows them to generate rapid Ca^2+^ signals, but this aspect makes them more sensitive to excitotoxicity, that in turn could promote motoneuron disease [61].

Due to the lack of data, extensive study of the kinetics, the affinity and calcium binding capacity of the presented CaBPs are beyond the scope of our review. However, considering that there is a strong correlation between the calcium-binding properties of certain CaBPs and the electrophysiological and metabolic behaviour of the neurons that express them, further studies are necessary to better understand the functions of these proteins.

## Figures and Tables

**Table 1:** Putative functions of calcium-binding proteins (CaBPs) involved in neuronal signalling in the central nervous system (CNS).

Gene	Complete Name (Alternative Name)	Gene Ontology/CNS Function(s)
Cabp1	Ca^2+^-binding protein 1, (Caldendrin)	Modulator of Ca^2+^ channel activity; Ca^2+^ sensor; fine-tuning of CaV.1/2
Cabp4	Ca^2+^ -binding protein 4	Modulator of Ca^2+^ channel activity
Cabp5	Ca^2+^-binding protein 5	Modulator of Ca^2+^ channel activity
Cabp7	Ca^2+^-binding protein 7, (Calneuron-2)	Modulator of Ca^2+^ channel activity
Calb1	Calbindin D-28 K	Ca^2+^ sensor/buffer activity
Calb2	Calretinin, (Calbindin 2)	Ca^2+^ sensor/buffer activity
Calm1	Calmodulin 1	Ca^2+^ sensor/Ca^2+^ signalling
Calm2	Calmodulin 2	Ca^2+^ sensor/Ca^2+^ signalling
Calm3	Calmodulin 3	Ca^2+^ sensor/Ca^2+^ signalling
Calu	Calumenin (Crocalbin)	Unknown
Cgref1	Cell growth regulator with EF-hand domain 1	Unknown
Chp1	Calcineurin-like EF-hand protein 1, (1500003O03Rik RIKEN cDNA 1500003O03 gene)	Unknown
Cib2	Ca^2+^- and integrin-binding family member 2 (Calmyrin 2, Kip2)	Unknown
Efcab1	EF-hand Ca^2+^-binding domain 1	Unknown
Efcab4a	EF-hand Ca^2+^-binding domain 4A, (Cracr2b)	Ca^2+^ sensor activity
Efcab12	EF-hand Ca^2+^-binding domain 12 (BC060267)	Unknown
Efcab14	EF-hand calcium binding domain 14, (4732418C07Rik, RIKEN cDNA 4732418C07 gene, Kiaa0494)	Unknown
Efhb	EF-hand domain family member B	Unknown
Fstl4	Follistatin-like 4 (Spig1)	Unknown; negative regulator of BDNF maturation
Fstl5	Follistatin-like 5	Unknown
Guca1b	Guanylate cyclase activator 1B (Gcap2, Rp48)	Ca^2+^-sensitive guanylate cyclase activator activity, Ca^2+^ sensor activity
Hpca	Hippocalcin	Ca^2+^ sensor activity
Hpcal1	Hippocalcin-like 1 (Vilip3, Visinin-like protein 3)	Ca^2+^ sensor activity
Hpcal4	Hippocalcin-like 4 (Vilip2, Neural visinin-like protein 2)	Ca^2+^ sensor activity
Kcnip1	kV channel-interacting protein 1 (Kchip1)	Ca^2+^ sensor activity; modulation of Kv4 activity/control of neuronal excitability
Kcnip2	kV channel-interacting protein 2 (Kchip2)	Ca^2+^ sensor activity; modulation of Kv4 activity/control of neuronal excitability
Kcnip4	kV channel-interacting protein 4 (Kchip4, Calp)	Ca^2+^ sensor activity; modulation of Kv4 activity/control of neuronal excitability
Mcfd2	Multiple coagulation factor deficiency 2 (F5F8D, LMAN1IP, SDNSF)	Unknown; survival factor for neural stem cells
Ncald	Neurocalcin delta	Ca^2+^ sensor activity
Ncs1	Neuronal Ca^2+^ sensor1 (frequenin homolog)	Ca^2+^ sensor activity; modulation of synaptic plasticity/neuronal secretion
Necab1	N-terminal EF-hand Ca^2+^-binding protein 1	Unknown
Necab2	N-terminal EF-hand Ca^2+^-binding protein 2	Unknown
Necab3	N-terminal EF-hand Ca^2+^-binding protein 3	Unknown
Pef1	Penta-EF-hand domain containing 1 (Peflin)	Unknown
Pkd2	Polycystic kidney disease 2 (PKD4, PC2, TRPP2)	Ca^2+^ channel activity
Plch1	Phospholipase C eta 1, (Kiaa1069)	Phosphoinositide phospholipase C activity; Ca^2+^ sensor activity
Prkcsh	Protein kinase C substrate 80 K-H, (Glucosidase 2 subunit beta)	Ca^2+^ sensor activity
Pvalb	Parvalbumin (Parvalbumin alpha)	Ca^2+^ sensor/buffer activity
Rcn1	Reticulocalbin 1 (Rcal)	Unknown
Rcn2	Reticulocalbin 2	Unknown
Rcvrn	Recoverin	Ca^2+^ buffer activity in phototransduction; Ca^2+^ sensor activity
Rhot1	Ras homolog gene family, member T1 (Miro1)	GTPase activity; Ca^2+^ sensor activity/mitochondrial trafficking in neurons
Rptn	Repetin	Unknown
Ryr1	Ryanodine receptor 1	Ryanodine-sensitive Ca^2+^-release channel activity
Ryr2	Ryanodine receptor 2, cardiac	Ryanodine-sensitive Ca^2+^-release channel activity
S100a9	S100 Ca^2+^-binding protein A9 (Calgranulin B)	Ca^2+^ buffer activity; neuro-inflammatory process
S100a10	S100 Ca^2+^-binding protein A10 (Calpactin)	Ca^2+^ buffer activity; serotonergic signalling
S100a11	S100 Ca^2+^-binding protein A11 (Calgizzarin)	Ca^2+^ buffer activity
S100a16	S100 Ca^2+^+-binding protein A16 (Protein S100F)	Ca^2+^ buffer activity
S100b	S100 protein, beta polypeptide, neural	Ca^2+^ buffer activity; biological marker of brain damage
Scgn	Secretagogin	Ca^2+^ sensor/buffer activity
Sdf4	Stromal cell-derived factor 4 (Cab45)	Unknown
Sri	Sorcin	Intracellular Ca^2+^ transport; modulator of ryanodine-sensitive Ca^2+^-release channel
Stim1	Stromal interaction molecule 1	Ca^2+^ sensor activity; Ca^2+^ signalling/storage/release
Stim2	Stromal interaction molecule 2 (Kiaa1482)	Ca^2+^ sensor activity
Tchhl1	Trichohyalin-like 1 (S100a17)	Unknown
Tesc	Tescalcin (Calcineurin-like protein 3)	Unknown
Vsnl1	Visinin-like 1 (Vilip-1, Hippocalcin-like protein 3)	Ca^2+^ sensor activity; regulator of receptors (P2X, glycine, nicotinic acetylcholine)
Zzef1	Zinc finger, ZZ-type with EF-hand domain 1, (Kiaa0399)	Unknown

**Table 2:** Putative functions of calcium-binding proteins with lower expression in the central nervous system based on the Girard database.

Gene	Complete Name (Alternative Name)	Gene Ontology/CNS Function(s)
Ankrd5	Ankyrin repeat domain 5 (Ankef1)	Unknown
Caln1	Calneuron 1	Ca^2+^-buffer/sensor activity
Ccdc48	Coiled-coil domain containing 48 (Efcc1, EF-hand and coiled-coil domain containing 1)	Unknown
Chp2	Calcineurin B homologous protein 2 (2010110p09rik, RIKEN cDNA 2010110P09 gene)	Unknown
Efcab2	EF-hand Ca^2+^-binding domain 2	Unknown
Efcab5	EF-hand Ca^2+^-binding domain 5	Unknown
Efcab6	EF-hand Ca^2+^-binding domain 6	Unknown
Efcab7	EF-hand Ca^2+^-binding domain 7	Unknown
Efcab9	EF-hand Ca^2+^-binding domain 9	Unknown
Efcab4b	EF-hand Ca^2+^-binding domain 4B (CRACR2A)	Unknown
Efcab10	EF-hand Ca^2+^-binding domain 10	Unknown
Efcab11	EF-hand Ca^2+^-binding domain 11 (Egfem1, EGF-like and EMI domain containing 1)	Unknown
Efhc1	EF-hand domain (C-terminal) containing 1	Unknown
Efhc2	EF-hand domain (C-terminal) containing 2	Unknown
Efhd1	EF-hand domain containing 1	Unknown
Guca1a	Guanylate cyclase activator 1a	Ca^2+^-sensitive guanylate cyclase activator activity, Ca^2+^ sensor activity
Pkd2l1	Polycystic kidney disease 2-like 1	Cation channel activity
Rcn3	Reticulocalbin 3	Unknown
S100a1	S100 Ca^2+^-binding protein A1	Ca^2+^-buffer activity
S100a3	S100 Ca^2+^-binding protein A3	Ca^2+^-buffer activity
S100a4	S100 Ca^2+^-binding protein A4	Ca^2+^-buffer activity
S100a5	S100 Ca^2+^-binding protein A5	Ca^2+^-buffer activity
S100a6	S100 Ca^2+^-binding protein A6 (Calcyclin)	Ca^2+^-buffer activity
S100a7a	100 Ca^2+^-binding protein A7A	Ca^2+^-buffer activity
S100a8	S100 Ca^2+^-binding protein A8 (Calgranulin A)	Ca^2+^-buffer activity
S100a13	S100 Ca^2+^-binding protein A13	Ca^2+^-buffer activity
S100a14	S100 Ca^2+^-binding protein A14	Ca^2+^-buffer activity
Synrg	Synergin, gamma	Unknown
1700109H08Rik	RIKEN cDNA 1700109H08 gene	Unknown

## Data Availability

Publicly available datasets were analyzed in this study (www.proteinatlas.org accessed in 29 April 2020, www.brainrnaseq.org accessed in 12 May 2020). The compiled databases presented in the article are available as Supplementary Materials.

## Supplementary Materials

The following are available online at https://www.mdpi.com/article/10.3390/brainsci11050634/s1, Table S1: Numerical data of regional expression levels of the selected CaBP genes. Mouse gene data from Human Protein Atlas (www.proteinatlas.org, accessed on 29 April 2020) transcriptomics analysis is shown as NX values in different brain regions. Heat map format is presented in Figure 3; Table S2: Numerical data of transcript expression levels in 10 brain regions based on in situ hybridisation. RNA Allen mouse brain region gene data of the selected CaBPs from ISH analysis is represented by the expression energy value. Heat map format is presented in Figure 4; Table S3: Numerical data of cell-type specific gene expression levels of selected CaBPs from the Brain RNA-seq database (www.brainrnaseq.org, accessed on 12 May 2020). Expression level estimation is reported as fragments per kilobase of transcript sequence per million mapped fragments (FPKM). Heat map format is presented in Figure 5; Table S4: Numerical data of mouse regional gene data of CaBPs with lower expression in the CNS by Girard database. Transcriptomics analysis from Human Protein Atlas (www.proteinatlas.org, accessed on 29 April 2020) showing NX values of genes in different brain regions. Heat map format is presented in Figure 6; Table S5: Numerical data of transcript expression levels of CaBPs with lower expression in the CNS by Girard database in 10 brain regions, based on in situ hybridisation. RNA Allen mouse brain region gene data is represented by the expression energy value. Heat map format is presented in Figure 7; Table S6: Numerical data of cell-type specific gene expression levels of CaBPs with lower expression in the CNS by Girard database. Expression level estimation from Brain RNA-seq database (www.brainrnaseq.org, accessed on 12 May 2020) is reported as fragments per kilobase of transcript sequence per million mapped fragments (FPKM). The heat map format is presented in Figure 8.
