# Supplementary material for: New Approach for Untangling the Role of Uncommon Calcium-Binding Proteins in the Central Nervous System
Source: Brain Sci. 2021 May 14;11(5):634. doi: 10.3390/brainsci11050634 (PMC8156796; doi:10.3390/brainsci11050634)
Supplement: Supplementary file 1 [file brainsci-11-00634-s001.zip › brainsci-1203654-supplementary/Legends for Supplementary Materials.pdf]

New approach for untangling the role of uncommon calcium-binding proteins in the central nervous system

Krisztina Kelemen and Tibor Szilágyi

Online Supplement

Supplemental Table S1. Numerical data of regional expression levels of the selected CaBP genes. Mouse gene data from Human Protein Atlas (www.proteinatlas.org) transcriptomics analysis is shown as NX values in different brain regions. Heat map format is presented in Figure 3.

In this dataset, transcript expression levels are summarized per gene in 13 brain regions based on RNA-seq transcripts per million (TPM) and protein-coding transcripts per million (pTPM). The TPM values were TMM (trimmed means of M values) normalized between all samples respectively, and then each gene was Pareto scaled. The normalized expression (NX) calculation is based on pTPM of the individual samples. The Human Protein Atlas version 19.3 and Ensembl version 92.38 were used for analysis.

Supplemental Table S2. Numerical data of transcript expression levels in 10 brain regions based on in situ hybridisation. RNA Allen mouse brain region gene data of the selected CaBPs from ISH analysis is represented by the expression energy value. Heat map format is presented in Figure 4.

The expression energy based on ISH, is defined as the sum of expression pixel intensity divided by the sum of all pixels in division.

Supplemental Table S3. Numerical data of cell-type specific gene expression levels of selected CaBPs from the Brain RNA-seq database (www.brainrnaseq.org). Expression level estimation is reported as fragments per kilobase of transcript sequence per million mapped fragments (FPKM). Heat map format is presented in Figure 5.

Astro: astrocytes, OPC: oligodendrocyte progenitor cells, NFO: newly formed oligodendrocytes, Myelinating: myelinating oligodendrocyte, MGL: microglia/macrophage, Endo: endothelial.

Supplemental Table S4. Numerical data of mouse regional gene data of CaBPs with lower expression in the CNS by Girard database. Transcriptomics analysis from

Human Protein Atlas (www.proteinatlas.org) showing NX values of genes in different brain regions. Heat map format is presented in Figure 6.

In this dataset, transcript expression levels are summarized per gene in 13 brain regions based on RNA-seq transcripts per million (TPM) and protein-coding transcripts per million (pTPM). The TPM values were TMM (trimmed means of M values) normalized between all samples respectively, and then each gene was Pareto scaled. The normalized expression (NX) calculation is based on pTPM of the individual samples. The Human Protein Atlas version 19.3 and Ensembl version 92.38 were used for analysis.

Supplemental Table S5. Numerical data of transcript expression levels of CaBPs with lower expression in the CNS by Girard database in 10 brain regions, based on in situ hybridisation. RNA Allen mouse brain region gene data is represented by the expression energy value. Heat map format is presented in Figure 7.

The expression energy based on ISH, is defined as the sum of expression pixel intensity divided by the sum of all pixels in division

Supplemental Table S6. Numerical data of cell-type specific gene expression levels of CaBPs with lower expression in the CNS by Girard database. Expression level estimation from Brain RNA-seq database (www.brainrnaseq.org) is reported as fragments per kilobase of transcript sequence per million mapped fragments (FPKM). Heat map format is presented in Figure 8.

Astro: astrocytes, OPC: oligodendrocyte progenitor cells, NFO: newly formed oligodendrocytes, Myelinating: myelinating oligodendrocyte, MGL: microglia/macrophage, Endo: endothelial.
